# Case Report: Catheter ablation of atrial fibrillation in a patient with cor triatriatum sinistrum: the pivotal role of intracardiac echocardiography and electroanatomic mapping

**DOI:** 10.3389/fcvm.2026.1777314

**Published:** 2026-05-26

**Authors:** Mingjie Lin, Nan Yang, Jing Kong, Jingquan Zhong, Kai Zhang

**Affiliations:** State Key Laboratory for Innovation and Transformation of Luobing Theory, Key Laboratory of Cardiovascular Remodeling and Function Research of Chinese Ministry of Education, Chinese National Health Commission, Chinese Academy of Medical Sciences and Shandong Province, Department of Cardiology, Qilu Hospital of Shandong University, Jinan/Qingdao, China

**Keywords:** atrial fibrillation, catheter ablation, cor triatriatum sinister, electroanatomic mapping, intracardiac echocardiography

## Abstract

Cor triatriatum sinister (CTS) is a rare congenital cardiac anomaly that presents significant challenges for catheter ablation of atrial fibrillation (AF) due to its complex left atrial anatomy. We report the case of a 47-year-old man with persistent AF and CTS who successfully underwent radiofrequency catheter ablation. Intracardiac echocardiography was critical in guiding transseptal puncture and delineating the atrial chambers, while electroanatomic mapping revealed a high-voltage area rather than a low-voltage zone at the fibromuscular membrane. This case underscores the importance of advanced imaging and individualized ablation strategies in managing arrhythmias in structurally abnormal hearts.

## 1 Introduction

Cor triatriatum sinister (CTS) is an uncommon congenital heart defect, accounting for approximately 0.1%–0.4% of all congenital cardiac anomalies, with a slightly higher prevalence in men (1, 2). It is characterized by division of the left atrium into two chambers by a fibromuscular membrane: a posterosuperior accessory chamber that receives the pulmonary veins and an anteroinferior true chamber containing the left atrial appendage and mitral valve (3). The embryological origin of CTS remains debated, with prevailing theories suggesting either failure of incorporation of the common pulmonary vein into the left atrial wall or abnormal development of the septum primum (4). The clinical presentation of CTS varies depending on the degree of obstruction at the membrane fenestration. While symptomatic cases often manifest in infancy, adults may present with atrial fibrillation (AF)—the most common arrhythmia in this population, occurring in about one-third of cases (1). The arrhythmogenic substrate in CTS is thought to arise from atrial remodeling due to chronic pressure overload, anatomical distortion, and the potential for myocardial fibers within the membrane itself to facilitate reentry (5, 6).

Catheter ablation (CA) has emerged as a viable therapeutic option for AF in patients with CTS (3, 7–9). However, the procedure is fraught with challenges, including difficult transseptal access, catheter navigation around the membrane, and substrate modification in an altered anatomical landscape (5, 6). Since the first reported case of AF ablation in CTS by Yamada et al. (10), fewer than 20 cases have been described in the literature, predominantly using radiofrequency ablation guided by transesophageal echocardiography or intracardiac echocardiography (ICE) (5). ICE, in particular, has proven indispensable for real-time anatomical visualization, safe transseptal puncture (TSP), and ensuring adequate catheter contact (11).

In this report, we present a case of persistent AF ablation in a patient with CTS, emphasizing the utility of ICE in overcoming procedural challenges and the insights gained from electroanatomic mapping of the accessory chamber. Our findings contribute to the growing evidence on optimizing ablation strategies for complex congenital heart disease.

## 2 Case presentation

**Figure 1:**
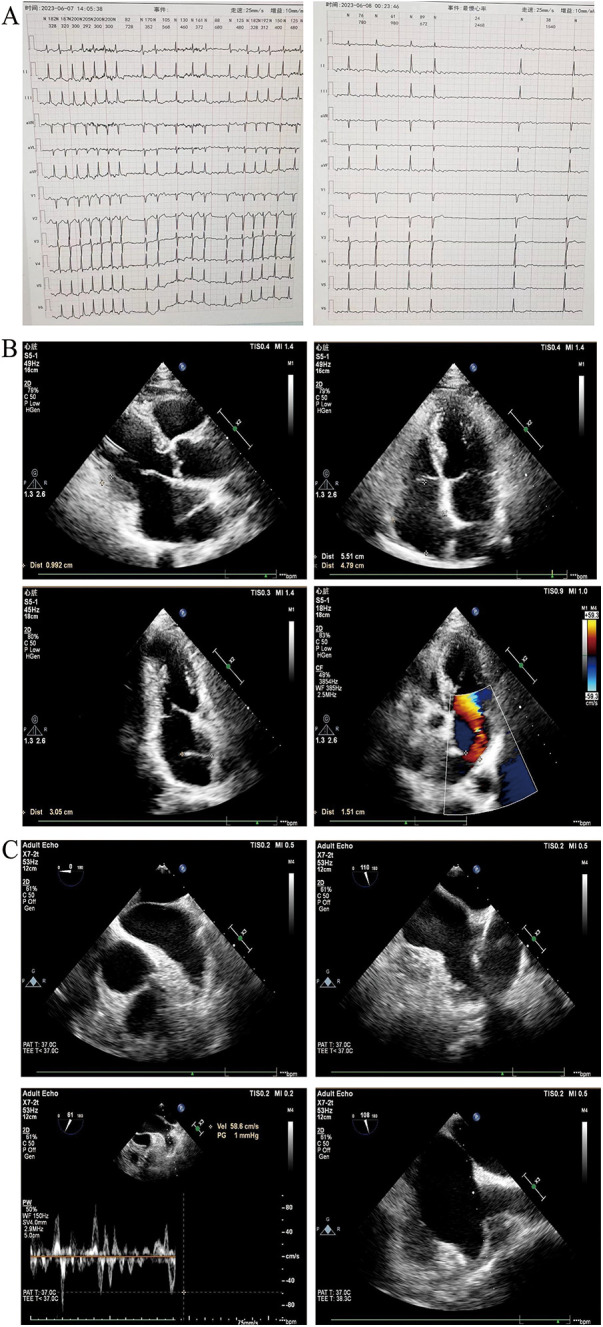
Preprocedural evaluations. **(A)**​ Preprocedural 24-h Holter monitoring demonstrated persistent atrial fibrillation with a prolonged RR interval up to 2.47 s. Coarse fibrillatory waves are visible on the surface electrocardiogram. **(B)**​ Transthoracic echocardiogram views revealed cor triatriatum sinister. The fibromuscular membrane is located at the midportion of the left atrium, with a fenestration orifice measuring approximately 15.1 mm and a membrane length of 30.5 mm (consistent with Type III CTS). **(C)**​ Preprocedural transesophageal echocardiography confirmed the absence of intracardiac thrombi.

A 47-year-old man presented with a 1-year history of palpitations triggered by hunger or exertion, which had intensified over the preceding two months. Episodes were accompanied by chest tightness and pain but no syncope. External electrocardiogram (ECG) confirmed AF, and Holter monitoring confirmed persistent episodes and pauses up to 2.47 s (Figure 1A). The patient had a history of hypertension controlled with medication (CHA_2_DS_2_-VA score 1). Transthoracic echocardiography revealed a dilated left atrium (47 mm) and right atrium, with a membrane dividing it into two chambers. Volumetric assessment was not performed as part of the routine preablation workup at our center for this case. The membrane measured 30.5 mm with a 15.1-mm fenestration orifice (>1.0 cm, Type III CTS) (Figure 1B) (3). Transesophageal ultrasound ruled out the formation of blood clots in the atria (Figure 1C).

**Figure 2:**
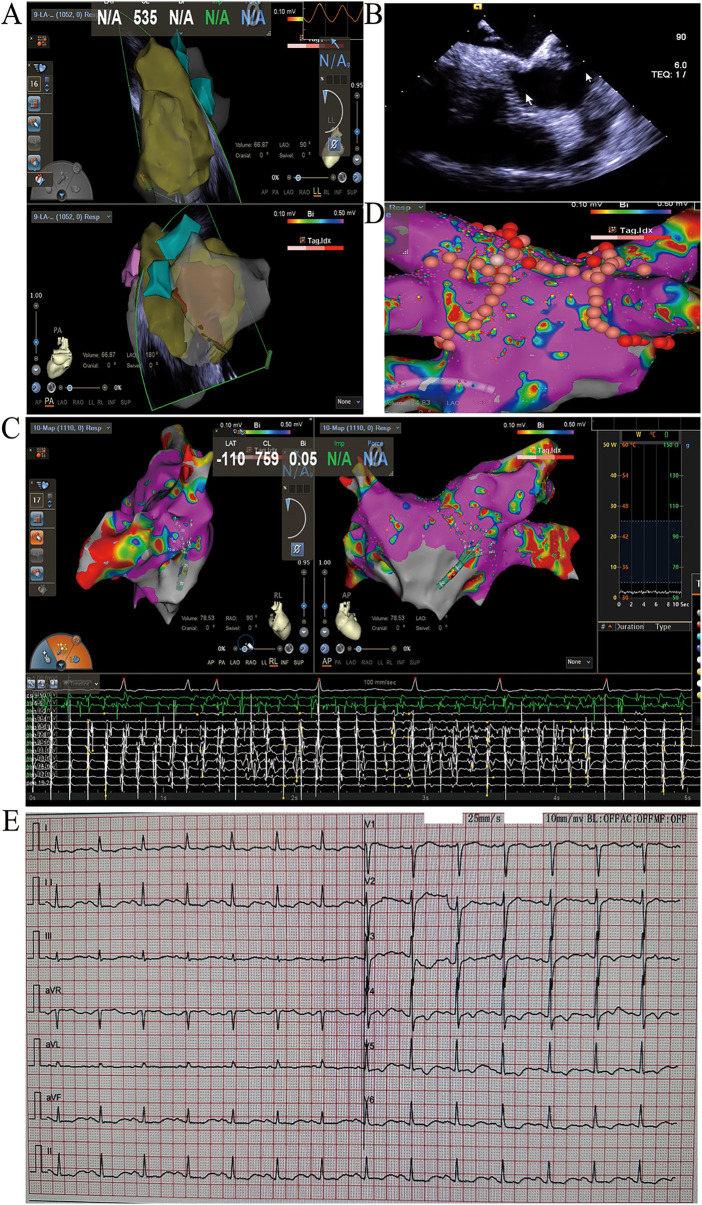
Intraprocedural findings and postablation follow-up.​ **(A)** left atrial geometry reconstructed using intracardiac echocardiography (ICE) integrated with the CARTO 3 system, illustrating the accessory chamber (gray), true atrium (green), and fibromuscular membrane (brown). **(B)** Intraprocedural ICE imaging revealed a thickened interatrial septum with the membrane located at its mid-portion. **(C)** Electroanatomic voltage mapping demonstrated bilateral pulmonary veins with two distinct branches each, preserved voltage in the membrane on the accessory chamber side. **(D)** Ablation lesions delivered following the CLOSE protocol principles (lesion spacing: 3–5 mm; ablation index targets: 350–400 for posterior walls, 450–480 for roof walls, and 480–520 for anterior walls). **(E)** A circular mapping catheter advanced through the membrane fenestration into the true atrium, confirming preserved anterior wall voltage. **(F)** At 30-month follow-up, electrocardiogram showed no recurrence of atrial tachycardia.

The CARTO-Sound module (Biosense Webster, Inc., Irvine, CA, USA) enables the integration of ICE with 3D electroanatomic mapping, providing a powerful tool for delineating complex anatomy. The accessory chamber—which typically receives all four pulmonary veins—and the true chamber—containing the left atrial appendage and mitral valve—were mapped separately (3). After introducing the ICE catheter into the right atrium, the true atrial chamber, accessory chamber, and fibromuscular membrane region were delineated and different color schemes were applied to differentiate the two chambers and the membrane, facilitating spatial orientation during catheter navigation (Figure 2A and Supplementary Video 1). As observed in the procedure (Figure 2B and Supplementary Video 2), the septum in CTS patients is thickened, complicating conventional puncture techniques. Under continuous ICE and X-ray guidance, the puncture needle was advanced from the superior vena cava to the posterior middle part of the atrial septum.

The characteristic “tenting” sign was clearly visualized, confirming adequate needle contact and pressure application. The initial puncture inadvertently accessed the true atrial chamber (verified by contrast injection and ICE). After repositioning, three attempts were made. The critical breakthrough occurred when the needle was repositioned to a more posterior and inferior location, directly targeting the accessory chamber. Successful entry was confirmed by ICE visualization of the needle tip and contrast injection opacifying the pulmonary veins within the accessory chamber (Supplementary Video 3). The entire TSP process—from the first attempt to final successful access into the accessory chamber—took approximately 15 min. Following successful TSP into the accessory chamber, detailed electroanatomic mapping was performed using a high-density PentaRay catheter (Biosense Webster). Mapping revealed the following key findings (Figure 2C and Supplementary Video 4): The atrial substrate within the accessory chamber demonstrated generally healthy myocardial tissue with preserved voltage characteristics. Both left and right pulmonary veins exhibited normal anatomical configurations, with no evidence of common pulmonary venous trunks. Mapping of the membrane on the accessory chamber side revealed a regular high-voltage area rather than a low-voltage zone, suggesting that the patient's membrane is not merely a fibrous sheet but a muscular membrane containing myocardial tissue capable of electrical activity. Based on these findings and the patient's relatively healthy atrial substrate, a targeted ablation approach was adopted. The strategy included pulmonary vein isolation (PVI) using wide-area circumferential ablation and additional roof line ablation to create a complete lesion set. Ablation was performed using a SmartTouch SF catheter (45–50W, 17 mL/min saline irrigation, less than 45°C, 5–15g contact force) according to CLOSE protocol principles (3–5 mm lesion space; AI 350–400 for posterior walls, 450–480 for roof walls, and 480–520 for anterior walls) (Figure 2D, Supplementary Figure 1). Following completion of the ablation lines, cardioversion at 150J successfully restored sinus rhythm. Complete PVI was confirmed by entrance and exit block. Bidirectional block across the roof line was also confirmed. Key procedure times were as follows: left atrium dwelling, approximately 90 min; radiofrequency delivery, 60 min; and total skin-to-skin time, 150 min.

Following the procedure, the patient was prescribed amiodarone (0.2 g, once daily) and rivaroxaban (15 mg, once daily) for 3 months. Follow-up was conducted via telephone clinics at 1, 3, 6, 12, and 30 months. At each contact, the patient reported complete resolution of palpitations and improved exercise tolerance. Rhythm monitoring included 12-lead ECGs at all follow-ups and 24-h Holter monitoring at 3 and 30 months. Antiarrhythmic and anticoagulant therapy was discontinued after the 3-month period. At the 30-month follow-up, ECG and 24-h Holter monitoring confirmed normal sinus rhythm with no AF recurrence (Figure 2E).

## 3 Discussion

This case highlights two critical aspects of CA for AF in patients with CTS: the indispensable role of advanced imaging in navigating complex anatomy and the evolving understanding of arrhythmogenic mechanisms in this rare congenital condition.

### 3.1 Navigating anatomical complexity: the pivotal role of ICE

This case highlights the indispensable role of ICE in navigating the unique anatomical challenges of CTS, principally the thickened interatrial septum that renders conventional transseptal puncture (TSP) techniques difficult and potentially hazardous. The choice of imaging guidance in AF ablation balances procedural safety, efficiency, and patient comfort. Although fluoroscopy provides real-time navigation, it offers only indirect, inferential anatomical information. Transesophageal echocardiography (TEE) provides high-resolution images and is the traditional standard for preprocedural left atrial appendage thrombus exclusion. However, TEE is intermittent, requires a separate operator, and is more invasive for the patient, often necessitating deeper sedation. In contrast, ICE provides continuous, real-time, and operator-controlled imaging from within the cardiac chambers.​ In our case, ICE was fundamental for the following: (1) preprocedural 3D anatomical reconstruction of the accessory chamber, true atrium, and the intervening membrane using the CARTO-Sound module; (2) real-time visualization of needle “tenting” and tissue deformation during TSP, allowing for immediate adjustment; and (3) definitive confirmation of successful access into the accessory chamber rather than the true chamber. Our experience is strongly supported by emerging high-level evidence. A recent large, multicenter randomized clinical trial directly comparing ICE to TEE for thrombus screening prior to AF ablation demonstrated that ICE was non-inferior to TEE in preventing periprocedural thromboembolic events. Importantly, the same trial found that ICE guidance was associated with significantly lower rates of major bleeding related to TSP, reduced fluoroscopy time, shorter preprocedural waiting times, and lower patient-reported anxiety (12). The synergy between real-time ICE visualization and electroanatomic mapping provides unparalleled anatomical guidance—a principle that our team has emphasized in the development of expert consensus guidelines on ICE utilization in complex cardiac procedures (11).

### 3.2 Electrophysiological mechanisms and ablation strategy in CTS

The pathogenesis of AF in CTS patients remains incompletely defined. Although the majority of reported cases involve persistent AF (approximately 70%), this does not necessarily indicate a diffusely abnormal atrial substrate in this population. As highlighted in the recent review by Bakytzhanuly et al. (3), successful rhythm control is often achieved with PVI alone or with limited additional substrate modification. This suggests that in many CTS patients, the arrhythmogenic focus may remain primarily pulmonary vein-driven, similar to paroxysmal AF in structurally normal hearts, rather than being solely a consequence of extensive atrial remodeling. In our case, the patient was a relatively young man with a short history of symptomatic AF (approximately 1 year) and preserved left atrial voltage on detailed electroanatomic mapping. In the absence of low-voltage zones or other indicators of a complex substrate, and given the primary goal of accessing the pulmonary veins within the accessory chamber, we adopted a tailored strategy. This consisted of PVI with the addition of a roof line—a common approach for persistent AF—followed by cardioversion. No further extensive linear lesions (e.g., mitral isthmus line) or ablation of complex fractionated electrograms was performed (13), as neither was indicated by the mapping findings. The 30-month success supports the feasibility of this anatomy-guided, substrate-informed approach for selected CTS patients with persistent AF. This observation suggests that the fibromuscular membrane itself may not serve as a primary arrhythmogenic substrate in most cases. Histological examinations have shown that the free membrane within the left atrium is predominantly composed of fibrous tissue, with muscular components typically confined to its junction with the atrial wall (5). In some cases, low-voltage zones adjacent to the membrane may represent areas of fibrotic remodeling secondary to turbulent flow or chronic pressure overload, potentially serving as arrhythmogenic foci. Notably, three reported cases have documented atrial tachycardia or flutter originating from the membrane region, necessitating additional linear ablation (5, 6). In contrast to these previous findings, our case presented a unique electrophysiological profile: The membrane in our patient was not identified as a low-voltage zone. This discrepancy may suggest that the electrophysiological properties of the membrane and its surrounding tissue in CTS are not uniform and could be influenced by factors such as the specific membrane composition, degree of mechanical stress, or chronicity of the condition. These findings underscore the importance of individualized substrate assessment and the potential need for more extensive ablation in select patients. Ultimately, the long-term success rates of AF ablation in CTS patients appear comparable to those in patients with structurally normal hearts, supporting the feasibility and efficacy of CA in this population (3, 5, 7–9).

## 4 Conclusion

CA of AF in patients with CTS is both feasible and effective when supported by meticulous preprocedural planning, real-time ICE guidance, and tailored substrate modification. Although most patients can be managed with PVI and limited substrate modification, a subset may require more extensive ablation based on individual electrophysiological characteristics. As clinical experience accumulates, continued reporting of such cases will refine our understanding of arrhythmia mechanisms and optimize procedural strategies in this challenging patient population.

## Data Availability

The datasets presented in this study are available upon request. Online videos can be founded in https://zenodo.org/records/19473912

## Supplementary material

SUPPLEMENTARY VIDEO 1 CARTO 3 in collaboration with ICE for left atrium model construction.

SUPPLEMENTARY VIDEO 2 ICE real-time monitoring for transseptal puncture.

SUPPLEMENTARY VIDEO 3 Left pulmonary vein and right pulmonary vein angiography.

SUPPLEMENTARY VIDEO 4 Electroanatomical mapping of the accessory chambers and membrane using the PentaRay catheter.

SUPPLEMENTARY FIGURE 1 Left lateral projection of the integrated electroanatomic map and ablation lesions.
