# Supplementary figures and images for: Case Report: Catheter ablation of atrial fibrillation in a patient with cor triatriatum sinistrum: the pivotal role of intracardiac echocardiography and electroanatomic mapping
Source: Front Cardiovasc Med. 2026 May 26;13:1777314. doi: 10.3389/fcvm.2026.1777314 (PMC13246362; doi:10.3389/fcvm.2026.1777314)

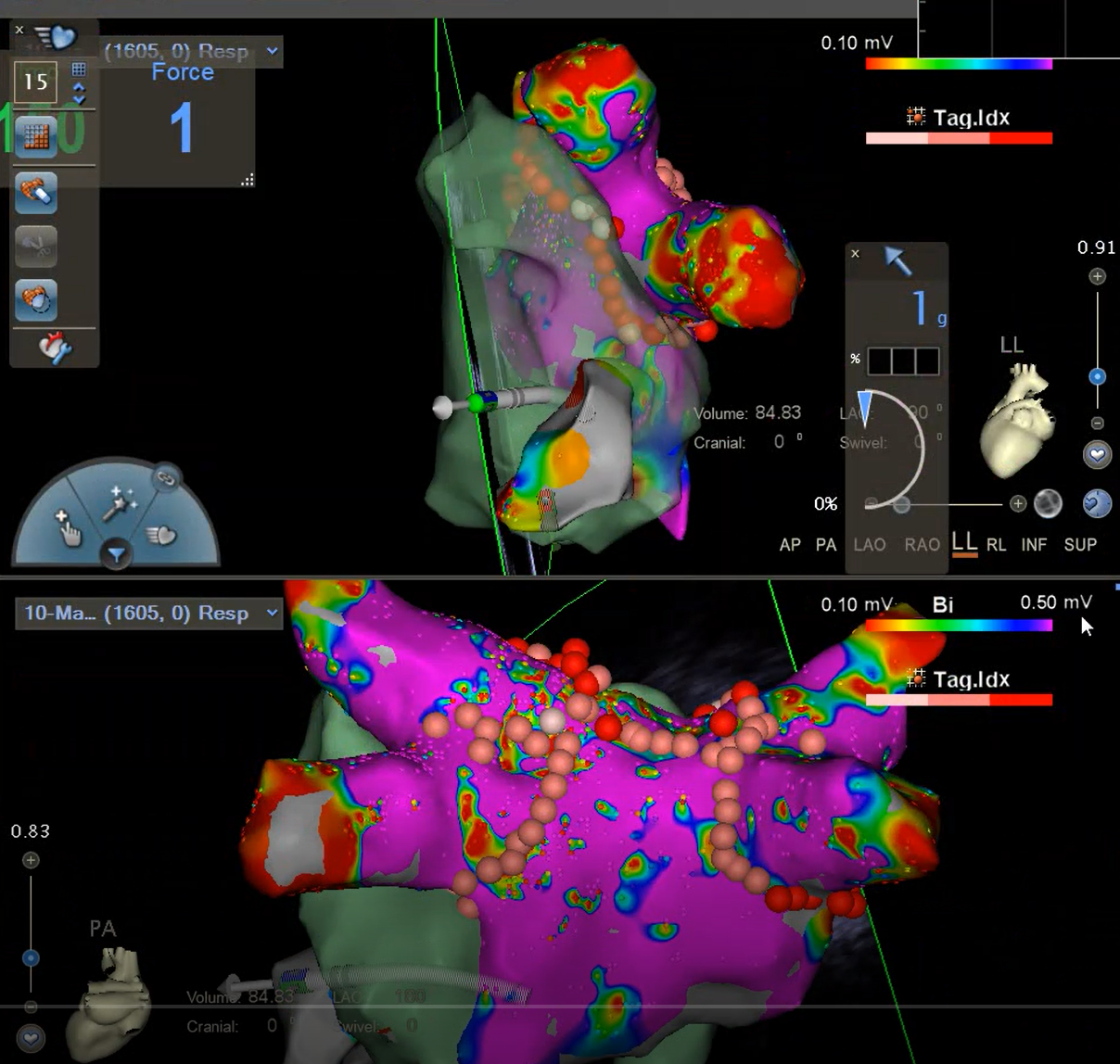

Supplement: SUPPLEMENTARY FIGURE 1 — Left lateral projection of the integrated electroanatomic map and ablation lesions. [file Image1.png]
